# miR-30a enhanced RIG-I-mediated type I interferon antiviral response by targeting USP14

**DOI:** 10.1128/spectrum.00188-25

**Published:** 2025-07-15

**Authors:** Jikai Zhang, Yiwen Wang, Ningye Sun, Botao Zou, Zijie Wang, Hang Yin, Jiaqian Xie, Banruo Xia, Nan Sun

**Affiliations:** 1 Jiangsu Key Laboratory of Immunity and Metabolism, Jiangsu International Laboratory of Immunity and Metabolism, Department of Pathogenic Biology and Immunology, School of Basic Medical Sciences, Xuzhou Medical University 38044 , Xuzhou, China; 2 Jiangsu Center for the Collaboration and Innovation of Cancer Biotherapy, Cancer Institute, Xuzhou Medical University 38044 , Xuzhou, China; Thomas Jefferson University, Philadelphia, Pennsylvania, USA

**Keywords:** miR-30a, RIG-I, USP14, ubiquitination, innate immunity, antiviral effects

## Abstract

Type I interferon (IFN) signaling plays a prominent role in the host innate immune defense against viral infection. The regulatory roles of miRNAs on the innate immune response remain to be further explored. Although miR-30a has been implicated in the regulation of various viral life cycles, the underlying mechanism on type I IFN signaling remains controversial. Herein, miR-30a was identified as a regulator of type 1 IFN production in macrophages. We observed that miR-30a expression was significantly decreased by vesicular stomatitis virus (VSV) or Sendai virus (SeV) infection in THP-1 cells. In return, overexpression of miR-30a promoted viral infection-triggered IFN and ISGs production to inhibit VSV or SeV replication. Mechanistically, miR-30a inhibited USP14 expression by binding with the 3′UTR of mRNA. USP14 was identified as an inhibitor of IFN signaling by removing the K63-linked ubiquitination from RIG-I, as previously reported. Consequently, miR-30a, by downregulating USP14 expression, enhanced the K63-linked ubiquitination of RIG-I to exert broad-spectrum antiviral effects. Overall, this study revealed a novel antiviral mechanism of miR-30a through the miR-30a-USP14-RIG-I axis and enriched miRNA–innate immunity regulatory networks.

**IMPORTANCE:**

miRNAs are involved in the regulation of innate immune responses and affect the life cycle of viruses. In this study, we identified miR-30a as a potent positive regulator of type I IFN signaling. The further mechanistic study revealed that miR-30a, by targeting and inhibiting USP14 expression, promoted RIG-I K63 ubiquitination to enhance type I IFN responses, thereby resulting in broad-spectrum antiviral effects against multiple viruses. As a complex regulatory network, the activation of type I interferon responses could subsequently reduce miR-30a expression to prevent the dysregulated activation. The insights gained could be crucial for developing innovative antiviral strategies to combat viral infections.

## INTRODUCTION

The innate immune response is initiated when host pattern-recognition receptors (PRRs) detect pathogen-associated molecular patterns (PAMPs) that are specifically expressed on the invading pathogens (1). As a result, PRRs initiate a sequence of signaling cascades that ultimately result in the production of type I interferons and pro-inflammatory cytokines (2, 3). Retinoic acid-inducible gene I (RIG-I)-like receptors (RLRs) are major PRRs that recognize RNA viruses and trigger antiviral responses, including RIG-I, melanoma differentiation-associated gene 5 (MDA5), and laboratory of genetics and physiology 2 (LGP2). RIG-I mainly recognizes short uncapped 5’-triphosphate (5’ppp) double-stranded RNA (dsRNA), while MDA5 interacts with long dsRNA (4, 5). LGP2 modulates RIG-I and MDA5 signaling rather than directly activating the IFN immune response (6). Under specific circumstances, RIG-I can participate in various physiological processes by sensing endogenous RNA, such as promoting therapeutic resistance and the expansion of breast cancer cells (7, 8). But the major physiological role of RIG-I is to recognize exogenous viral RNA stimulation and drive the production of type I interferon (IFN) and interferon-stimulated genes (ISGs), thereby triggering intracellular immune responses to defend viral infection (9). Therefore, the activity of RIG-I should be finely regulated to exert an appropriate protective immune response, thus avoiding excessive harmful autoimmune and inflammatory reactions.

MicroRNAs (miRNAs), as the non-coding RNAs with a length of approximately 22 nucleotides, silence target genes by degrading mRNAs or preventing protein translation. As an essential regulatory factor in the host, many studies have demonstrated that miRNAs could modulate the type I IFN signaling pathway, thereby affecting virus infection and replication. For example, miRNA-132-3p suppressed the type I IFN response by targeting interferon regulatory factor 1 (IRF1) to facilitate H1N1 influenza A virus (IAV) infection (10); miR-125a modulated IFN signaling and promoted hepatitis C virus (HCV) infection by targeting mitochondrial antiviral signaling protein (MAVS) and TNF receptor-associated factor 6 (TRAF6) (11). In this research, we focused on the regulatory roles of miR-30a on type I IFN signaling. Recent research indicates that miR-30a plays significant roles in numerous pathological processes, such as Alzheimer’s disease (12) and intrahepatic cholangiocarcinoma (13). However, as previously reported, miR-30a exerted various regulatory effects on type I IFN signaling by targeting different genes (14–17), indicating that the function of miR-30a varies across different cell types and viral infections. Aiming to elucidate the regulatory effects of miR-30a on innate immunity, we further explored the roles of miR-30a in type I IFN signaling in macrophages in this study. Consequently, miR-30a enhanced virus-triggered type I IFN immune response in THP-1 cells.

Protein functions are regulated by ubiquitination or deubiquitination, thereby affecting cellular protein degradation and signaling cascade transduction. Notably, RIG-I-mediated type I IFN antiviral signaling is also modulated by ubiquitination. The lysine 63 (K63)-linked polyubiquitination of RIG-I is essential for the activation and transduction of downstream signaling. Several studies have reported that RIG-I ubiquitination was regulated by some host factors, including E3 ubiquitin ligases, such as tripartite motif protein 25 (TRIM25) (18) and Riplet (also called RNF135) (19), as well as deubiquitinating enzymes (DUBs) like ubiquitin specific peptidases 21 (USP21) (20) and cylindromatosis (CYLD) (21). USPs represent the largest subfamily of DUBs and have been implicated in the regulation of the type I interferon antiviral response. In this study, we confirmed that miR-30a promoted RIG-I ubiquitination by directly targeting USP14, while USP14 was demonstrated to inhibit K63-linked ubiquitination of RIG-I, as previously reported (22). This research establishes a link among miR-30a, USP14, and RIG-I signaling. As noted in earlier studies, USP14 exhibits a dual role in regulating protein degradation; specifically, full-length USP14 or its UBL domain alone inhibits various proteasomal activities through DUB activity via an allosteric mechanism. On the other hand, USP14 promotes proteasomal degradation by activating the proteasome activities (23, 24). Besides, previous studies have also demonstrated that USP14 is involved in the occurrence and progression of certain cancers and neurodegenerative diseases (25, 26).

In this study, we observed that miR-30a enhanced RIG-I-mediated type I IFN immune responses in macrophages and identified USP14 as the target of miR-30a for the first time. Mechanistically, miR-30a, by downregulating USP14 expression, increased RIG-I K63-linked ubiquitination to promote IFN production to inhibit multiple RNA virus replications, including vesicular stomatitis virus (VSV) and Sendai virus (SeV). This study enriched miR-30a function in innate immunity and revealed a novel regulatory mechanism through the miR-30a-USP14-RIG-I ubiquitination axis, which is conducive to further elucidating the interaction between miRNAs and innate immunity.

## RESULTS

### Virus infection-triggered type I IFN signaling downregulates miR-30a expression in macrophages

**Fig 1:**
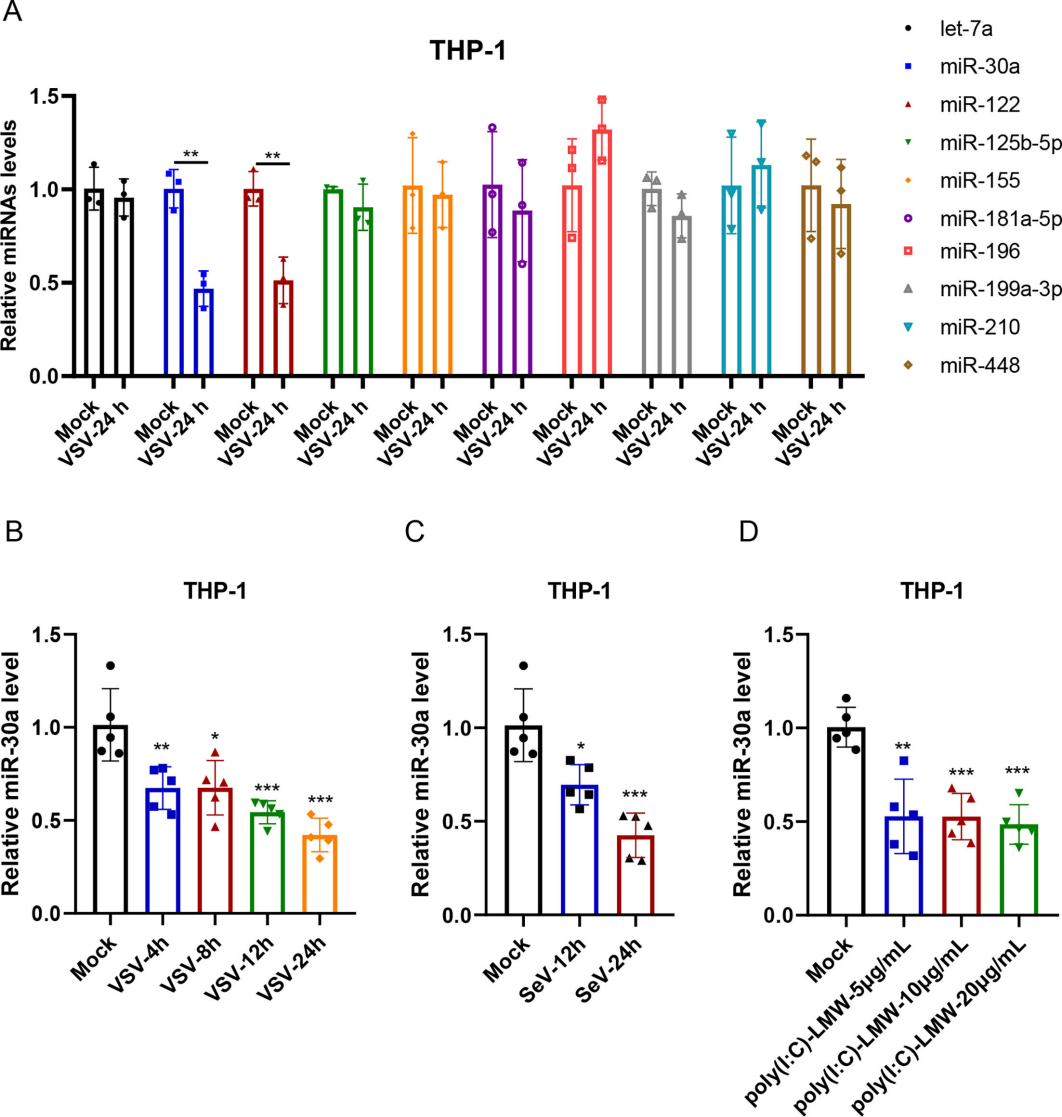
Viral infection-triggered activation of type I IFN antiviral signaling downregulates miR-30a expression. (**A**) THP-1 cells were infected with VSV at a MOI of 1. After 24 hours (h) post-infection, the expression of various cellular miRNAs was determined by stem-loop qRT-PCR. (**B and C**) THP-1 cells were inoculated with VSV (MOI = 1) (**B**) or SeV (100 HAU/mL) (**C**) for the indicated times, followed by determination of miR-30a expression via stem-loop qRT-PCR. (**D**) THP-1 cells were transfected with the indicated doses of poly(I:C)-LMW for 24 h. Then, the cellular miR-30a expression was detected by stem-loop qRT-PCR. Data are presented as mean ± SD and are representative of three independent experiments. The statistical significance was carried out using a two-tailed Student’s *t*-test. *P < 0.05, **P < 0.01, ***P < 0.001.

To identify miRNAs associated with the regulation of RIG-I-mediated innate immunity, several miRNAs were selected to analyze the expression in VSV-infected (an RNA virus sensed by RIG-I) THP-1 cells. As shown in Fig. 1A, we observe that both miR-122 and miR-30a were significantly decreased upon VSV infection. One previous study has demonstrated that miR-122 enhanced type I IFN expression by targeting the negative regulator suppressor of cytokine signaling 3 (SOCS3) (27). Besides, several studies have indicated that miR-30a exerted contradictory regulatory roles on type I IFN signaling upon various viral infections. Therefore, we aimed to further explore the regulatory roles of miR-30a upon type I IFN antiviral signaling in macrophages. Concretely, VSV infection remarked downregulated miR-30a expression at 4 h post-infection, reaching a minimum at 24 h in THP-1 cells (Fig. 1B). Similarly, SeV infection (another RNA virus recognized by RIG-I) also reduced miR-30a expression at 12 or 24 h post-infection (Fig. 1C). miR-30a expression was also decreased by poly(I:C) treatments in a dose-dependent manner (Fig. 1D). All of these results demonstrated that the activation of type I IFN antiviral signaling markedly downregulated miR-30a expression.

### miR-30a enhances VSV or SeV-induced type I IFN antiviral response to inhibit viral replication in macrophages

**Fig 2:**
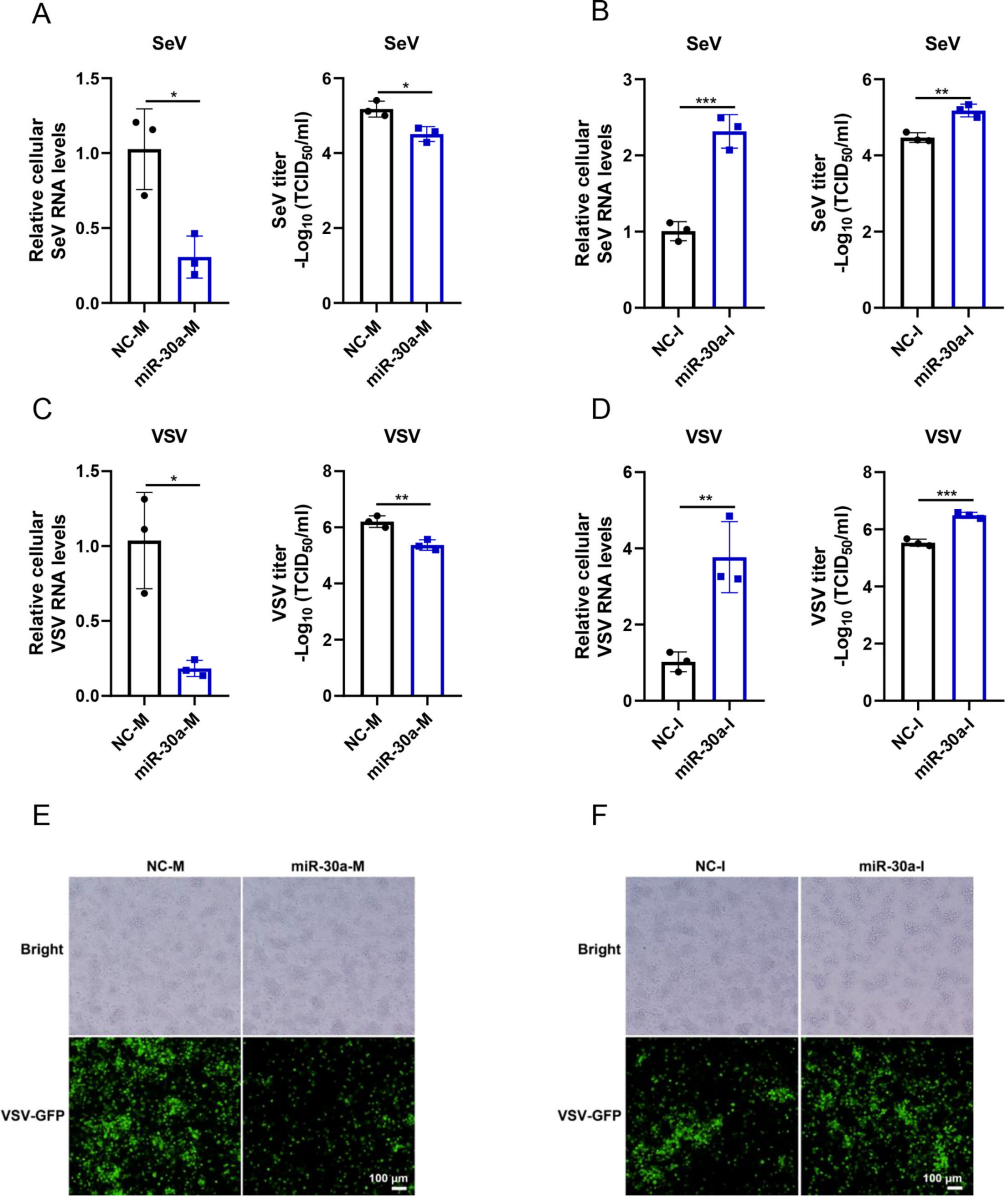
miR-30a potently suppresses VSV or SeV replication in macrophages. (**A and B**) Negative control mimics (NC-M) and miR-30a mimics (miR-30a-M) (**A**) or negative control inhibitors (NC-I) and miR-30a inhibitors (miR-30a-I) (**B**) were transfected into THP-1 cells. After SeV (100 HAU/mL) infection for 24 h, the cellular relative SeV RNA levels were detected by qRT-PCR, while virus titers in supernatants were determined via TCID_50_ assay. (**C to F**) Same as (**A and B**), but with VSV-GFP (MOI = 0.1) infection. The GFP fluorescence in VSV-infected cells was observed via fluorescence microscope (**E and F**). Data are presented as mean ± SD and are representative of three independent experiments. The statistical significance was carried out using a two-tailed Student’s *t*-test. *P < 0.05, **P < 0.01, ***P < 0.001.

Next, we aimed to investigate whether miR-30a expression changes affected the host antiviral response in macrophages. We overexpressed or knocked down cellular miR-30a via transfection of miRNA mimics or inhibitors, followed by VSV or SeV infection in THP-1 cells, respectively. Consequently, qRT-PCR or TCID_50_ assays showed that miR-30a overexpression significantly inhibited cellular SeV RNA levels as well as viral loads in supernatants (Fig. 2A), whereas knockdown of miR-30a promoted SeV replication in both cells and supernatants (Fig. 2B). Likewise, similar effects of miR-30a on the proliferation of VSV were observed (Fig. 2C and D). Moreover, GFP intensity observed by fluorescence microscopy also indicated that miR-30a overexpression and knockdown has opposite effects on VSV replications (Fig. 2E and F), suggesting that miR-30a exerts broad-spectrum antiviral functions in macrophages.

**Fig 3:**
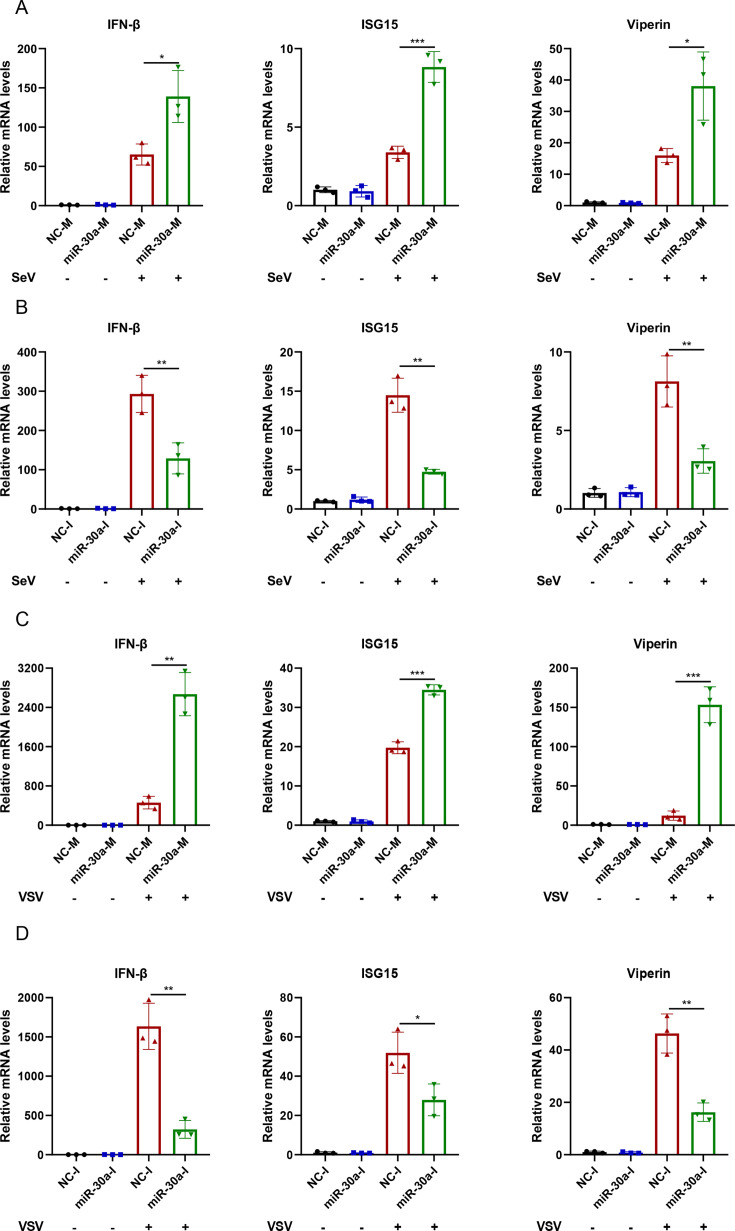
miR-30a markedly enhances SeV or VSV-triggered type I IFN antiviral response. (**A and B**) NC-M and miR-30a-M (**A**) , NC-I and miR-30a-I (**B**) were transfected into THP-1 cells for 36 h, followed by SeV infection (100 HAU/mL) for 12 h, respectively. Then, the relative mRNA expression levels of IFN-β and representative ISGs (ISG15 and Viperin) were determined via qPCR analysis. (**C and D**) Same as (**A and B**), but with VSV infection stimulation. Data are presented as mean ± SD and are representative of three independent experiments. The statistical significance was carried out using a two-tailed Student’s *t*-test. *P < 0.05, **P < 0.01, ***P < 0.001.

To demonstrate whether antiviral effects conferred by miR-30a occurred through the regulation of the type I IFN antiviral response, we further explored the regulatory roles of miR-30a on type I IFN signaling in macrophages. The results revealed that overexpression of miR-30a significantly enhanced (Fig. 3A), whereas knockdown of cellular miR-30a suppressed SeV-triggered IFN-β and representative ISG production (Fig. 3B). The similar results were also observed in the setting of VSV infection (Fig. 3C and D). All these results revealed that miR-30a exerts antiviral effects through positively regulating type I IFN antiviral response.

### miR-30a positively regulates type I IFN signaling by enhancing RIG-I K63-linked ubiquitination

**Fig 4:**
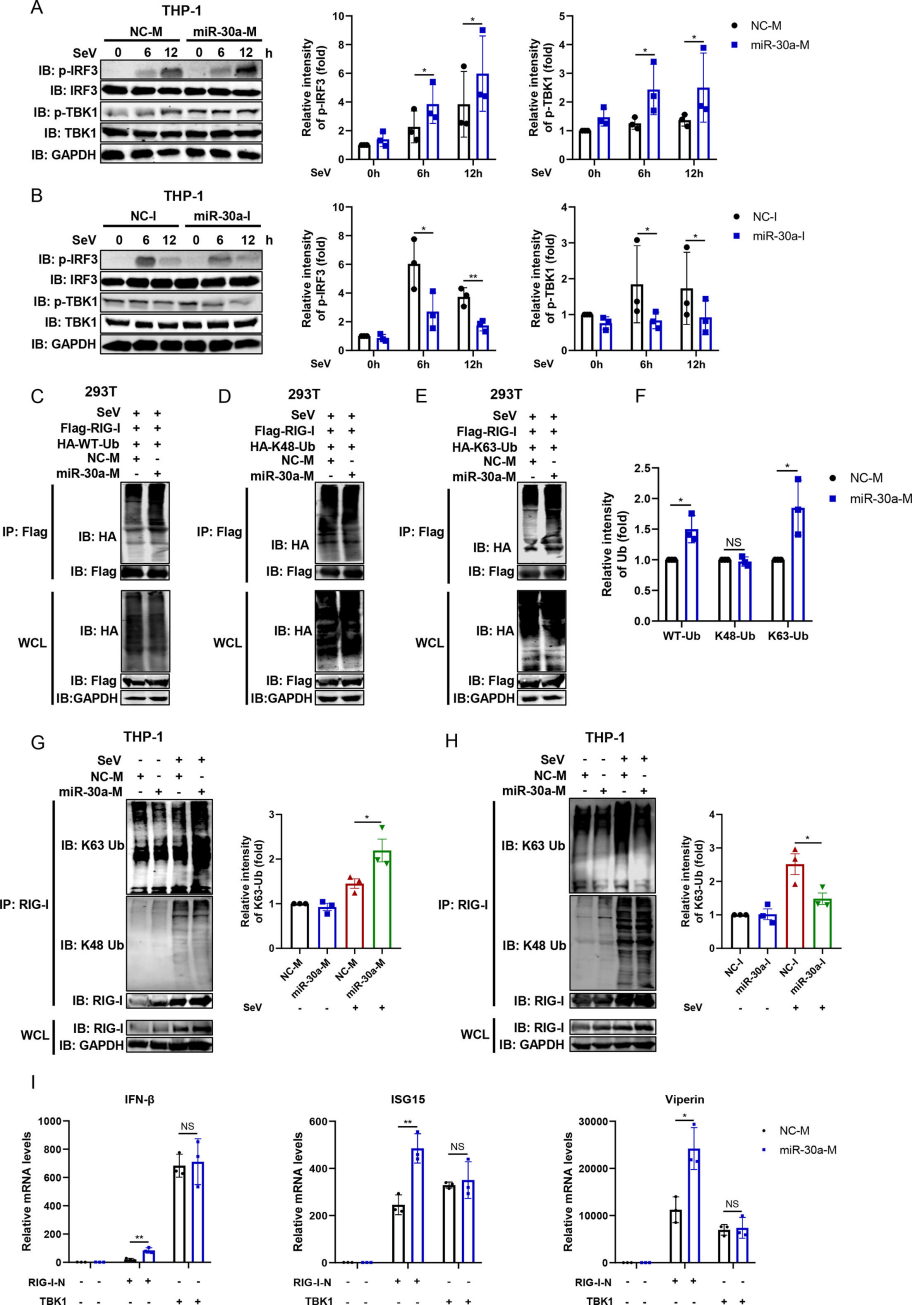
miR-30a enhances SeV-induced RIG-I signaling by increasing RIG-I K63-linked ubiquitination. (**A and B**) NC-M and miR-30a-M (**A**) or NC-I and miR-30a-I (**B**) were transfected into THP-1 cells for 36 h and inoculated with SeV (100 HAU/mL) for indicated times. Then, the cellular phosphorylated (p-) or total proteins of TBK1 and IRF3 were analyzed by immunoblotting using the specified antibodies. The relative intensities of p-IRF3 or p-TBK1 expression levels of **A** and **B** were quantified from three replicates using the Image Studio V5.2. (**C to E**) Flag-tagged RIG-I and HA-tagged Ub, including wild-type (WT) (**C**), K48 (**D**), and K63 (**E**), were co-transfected into HEK293T cells together with NC-M or miR-30a-M, respectively, followed by SeV (100 HAU/mL) infection for 12 h. Then, cell lysates were immunoprecipitated with anti-Flag antibodies. The exogenous ubiquitinated RIG-I was detected by immunoblot analysis. (**F**) The relative intensities of exogenous WT-Ub, K48-Ub, and K63-Ub expression levels from **C** to **E** were quantified from three replicates using the Image Studio V5.2. (**G and H**) NC-M and miR-30a-M (**G**) or NC-I and miR-30a-I (**H**) were transfected into THP-1 cells for 36 h and infected with SeV (100 HAU/mL) for 12 h. Then, the cell lysates were immunoprecipitated with anti-RIG-I antibodies, followed by immunoblot analysis of endogenous ubiquitinated RIG-I using anti-WT-Ub, anti-K48-Ub, or anti-K63-Ub, respectively. The relative intensities of endogenous K63-Ub expression levels of **G** and **H** were quantified from three replicates using the Image Studio V5.2. (**I**) NC or miR-30a mimics were co-transfected with RIG-I or TBK1 plasmids into HEK293T cells for 48 h. Then, the mRNA expression level of IFN-β and ISGs (ISG15 and Viperin) was determined by qRT-PCR. Data are presented as mean ± SD and are representative of three independent experiments. The statistical significance was carried out using a two-tailed Student’s *t*-test. *NS* >0.05, *P < 0.05, **P < 0.01.

Activation of RIG-I-mediated signaling leads to a series of downstream signaling cascades, including TANK-binding kinase 1 (TBK1) phosphorylation and subsequent phosphorylation and dimerization of interferon regulatory factor 3 (IRF3), thereby resulting in the production of IFN. Therefore, we assessed the impact of miR-30a on the RIG-I signaling cascades in THP-1 cells. Immunoblot analysis showed that miR-30a overexpression promoted SeV-triggered phosphorylation of TBK1 and IRF3 (Fig. 4A), while miR-30a inhibition blocked the phosphorylation level of TBK1 and IRF3 (Fig. 4B), indicating an enhanced signal transduction mediated by miR-30a upon RIG-I activation in macrophages. Earlier studies have established that the binding of K63-linked polyubiquitin chain to RIG-I was essential for the activation of RIG-I signaling. Therefore, the impact of miR-30a on the RIG-I ubiquitination was further identified. Consistent with the activating effects of phosphorylated TBK1 and p-IRF3, miR-30a significantly promoted exogenous SeV-triggered ubiquitination of RIG-I, specifically K63-linked, rather than K48-linked, ubiquitination (Fig. 4C to F). Similarly, miR-30a overexpression also enhanced endogenous K63-linked ubiquitination, whereas knockdown of miR-30a showed opposite effects on ubiquitinated RIG-I in THP-1 cells (Fig. 4G and H). Furthermore, miR-30a specifically enhanced type I IFN signaling activated by RIG-I overexpression rather than downstream TBK1 overexpression (Fig. 4I), indicating that miR-30a directly modulates RIG-I-mediated signaling. Collectively, we concluded that miR-30a, by promoting RIG-I K63-linked ubiquitination, resulted in an active IFN antiviral response.

### miR-30a targets USP14 3′UTR to inhibit its expression

**Fig 5:**
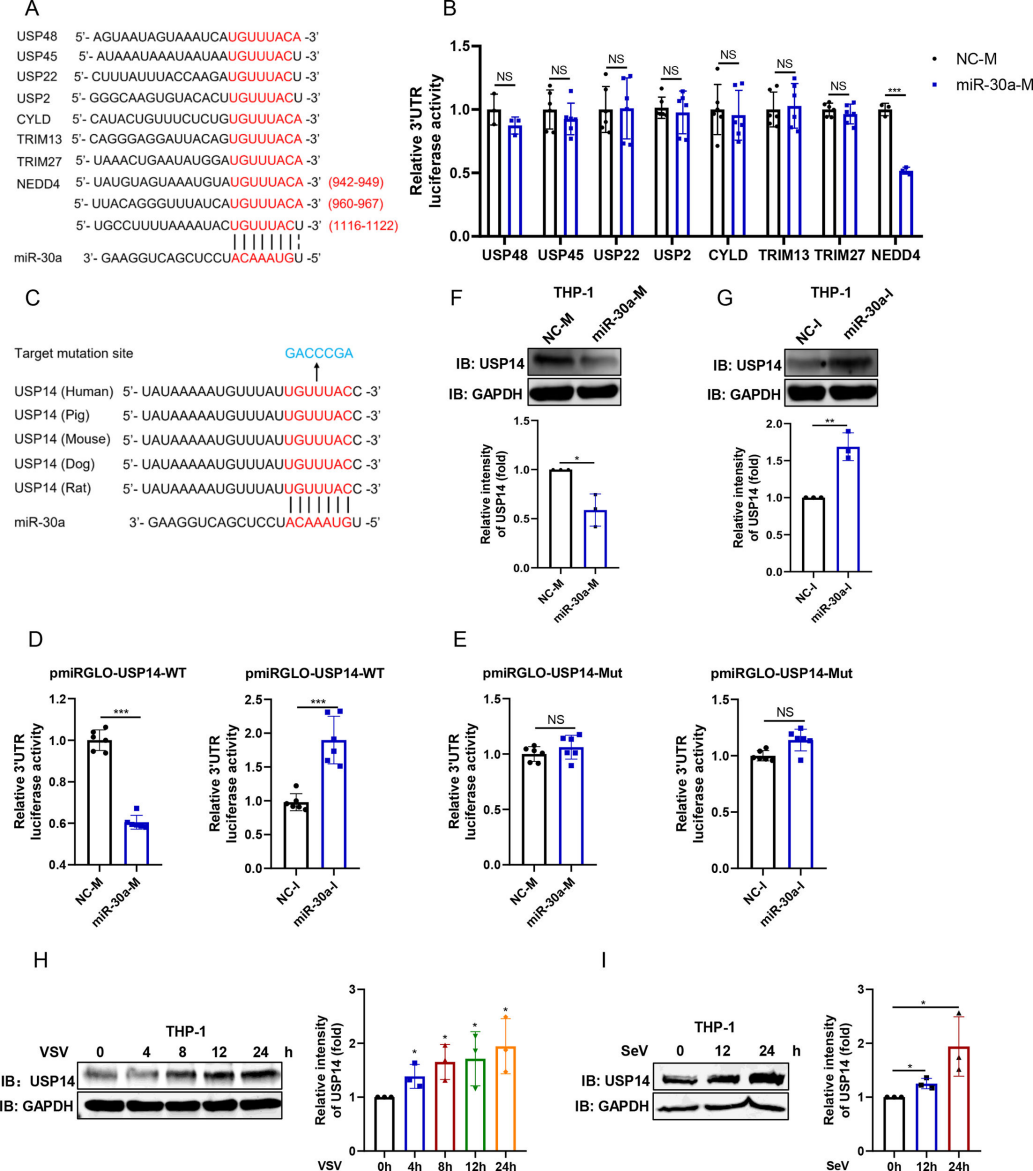
miR-30a specifically binds with USP14 3′UTR to inhibit its expression. (**A**) The putative binding sites between miR-30a seed region and 3′UTR of several predicted genes are illustrated. (**B**) The partial 3′UTR sequence of potential targets binding with miR-30a was cloned into pmiRGLO vectors. HEK293T cells were co-transfected with NC-M or miR-30a-M along with the corresponding 3′UTR reporter plasmids of the potential targets for 48 h. The relative luciferase activities were determined using dual-luciferase reporter (DLR) assays. (**C**) The putative binding sites between miR-30a seed region and wild-type (WT) or mutant (Mut) 3′UTR of USP14 were illustrated. (**D and E**) The WT (**D**) or Mut (**E**) 3′UTR reporter plasmids of USP14 were co-transfected with NC-M and miR-30a-M or NC-I and miR-30a-I into HEK293T cells for 48 h, respectively, followed by the measurement of relative luciferase activities. (**F and G**) NC-M and miR-30a-M (**F**) or NC-I and miR-30a-I (**G**) were transfected into THP-1 cells for 48 h. Then, the cellular USP14 expression was determined by immunoblot. The relative intensities of cellular USP14 expression levels of **F** and **G** were quantified from three replicates using Image Studio V5.2. (**H and I**) THP-1 cells were infected with VSV (MOI = 0.1) (**H**) or SeV (100 HAU/mL) (**I**) for the designated times, followed by immunoblot analysis of USP14 expression. The relative intensities of cellular USP14 expression levels of **H** and **I** were quantified from three replicates using the Image Studio V5.2. Data are presented as mean ± SD and are representative of three independent experiments. The statistical significance was carried out using a two-tailed Student’s *t*-test. *NS* >0.05, ***P < 0.001.

Aiming to dissect the mechanism of miR-30a regulating RIG-I-mediated signaling, we predicted the potential target genes whose 3′UTR bind to the seed region of miR-30a using the Targetscan online software (https://www.targetscan.org/vert_80/). Due to the impact of miR-30a on RIG-I ubiquitination, we selected several genes related to the ubiquitin regulation to identify the 3′-UTR luciferase activity using the luciferase reporter assay system, including USP family members (USP48, USP45, USP22, USP2, USP14, and CYLD) and E3 ubiquitin ligase (TRIM13 and TRIM27) (Fig. 5A and B). Consistent with the previous report, miR-30a significantly reduced the 3′-UTR luciferase activity of NEDD4 (16). Among these candidates, miR-30a overexpression significantly decreased, whereas miR-30a inhibition increased, the 3′-UTR luciferase activity of USP14 (Fig. 5D), which was identified as a regulator of RIG-I ubiquitination in a previous study (22). By contrast, when the binding region of USP14 3′UTR was mutated, as shown in Fig. 5C, miR-30a mimics or inhibitors had no effect on the luciferase activity (Fig. 5E). Consistently, USP14 protein expression was also decreased by miR-30a overexpression, while knockdown of miR-30a contributed to the accumulation of USP14 in THP-1 cells (Fig. 5F and G).

To further investigate the relationship between miR-30a and USP14, the protein levels of USP14 were also determined upon viral infection. As expected, in contrast with miR-30a expression (Fig. 1B to D), VSV or SeV infection markedly upregulated USP14 expression (Fig. 5H and I) in THP-1 cells. Taken together, these observations further demonstrated that miR-30a specifically targets USP14 3′UTR mRNA to inhibit its protein expression.

### USP14 impaired type I IFN antiviral response by eliminating K63-linked ubiquitination from RIG-I

**Fig 6:**
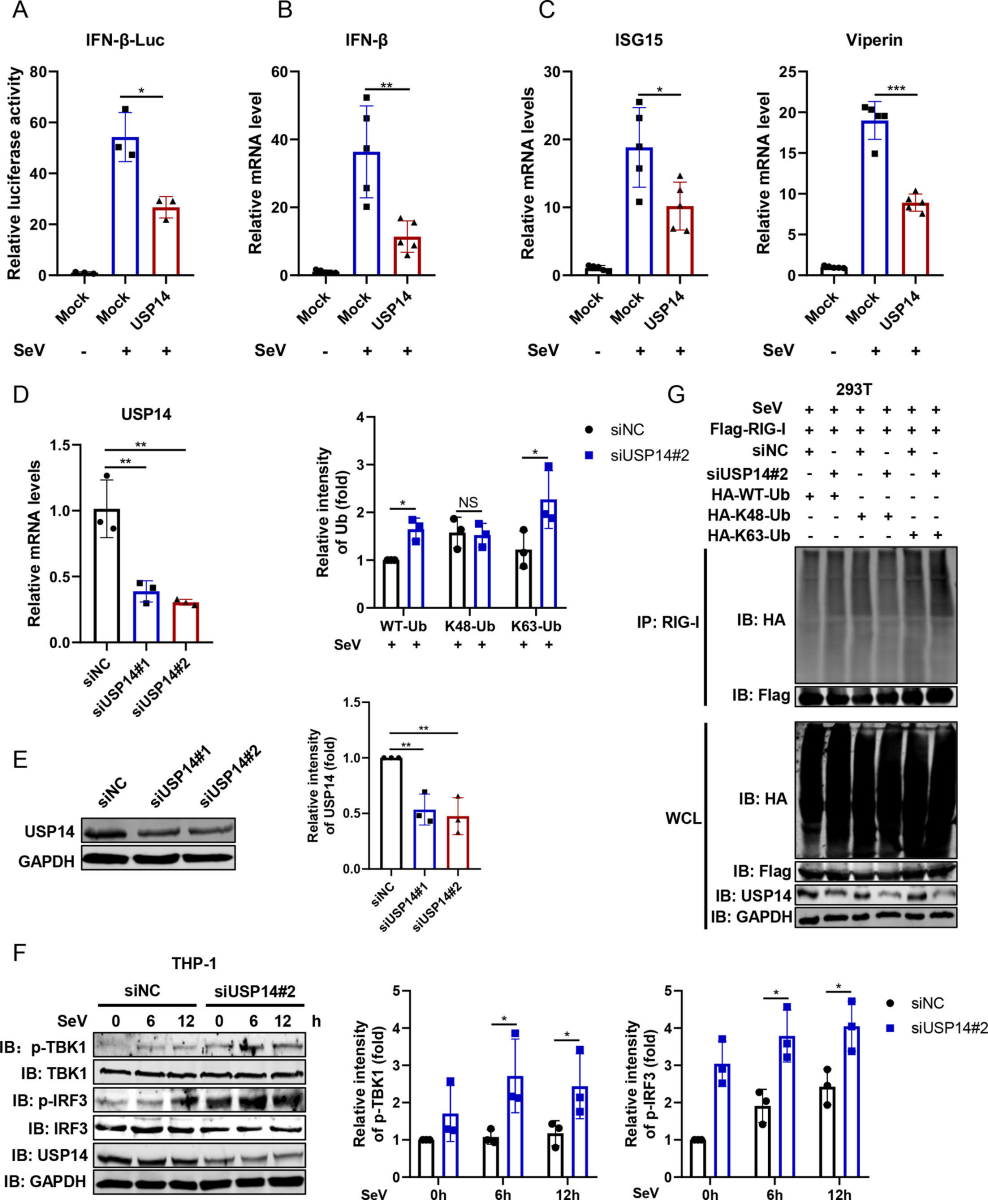
USP14 negatively regulates RIG-I-mediated type I IFN antiviral response. (**A**) HEK293T cells were co-transfected with the IFN-β-Luc promoter and pRL-TK plasmids, along with USP14 plasmid or empty vector for 36 h. Then, the cells were infected with SeV (100 HAU/mL) for 12 h, and the IFN-β promoter activity was assessed via DLR assays. (**B and C**) USP14 expression plasmid and empty vector were transfected into HEK293T cells for 36 h and infected with SeV (100 HAU/mL) for 12 h. Then, the relative mRNA levels of IFN-β (**B**) and representative ISGs (ISG15 and Viperin) (**C**) were determined by qRT-PCR analysis. (**D and E**) Two siRNAs targeting USP14 (siUSP14#1 and siUSP14#2) or negative control (siNC) were transfected into THP-1 cells for 48 h. The mRNA and protein levels of cellular USP14 were determined by qRT-PCR and immunoblot, respectively. The relative intensities of cellular USP14 expression levels of **E** were quantified from three replicates using Image Studio V5.2. (**F**) siNC or siUSP14#2 were transfected into THP-1 cells for 48 h and infected with SeV for 12 h. Then the cellular phosphorylated (p-) or total proteins of TBK1 and IRF3 were detected by immunoblot analysis using the indicated antibodies. The relative intensities of p-TBK1 or p-IRF3 expression levels were quantified from three replicates using the Image Studio V5.2. (**G**) Flag-RIG-I, HA-WT-Ub, K48-Ub, and K63-Ub were co-transfected into HEK293T cells together with siNC or siUSP14 for 48 h, respectively, followed by SeV infection for 12 h. The cell lysates underwent immunoprecipitation with anti-Flag antibodies, and exogenous ubiquitination of RIG-I was determined via immunoblot analysis. The relative intensities of exogenous WT-Ub, K48-Ub, and K63-Ub expression levels were quantified from three replicates using the Image Studio V5.2. Data are presented as mean ± SD and are representative of three independent experiments. The statistical significance was carried out using a two-tailed Student’s *t*-test. *P < 0.05, **P < 0.01, ***P < 0.001.

Given that miR-30a targets USP14, we proceeded to investigate how USP14 influences the regulation of type I IFN antiviral signaling. As shown in Fig. 6A through C, we observe that USP14 overexpression suppressed SeV-triggered IFN-β and downstream ISG production, suggesting that USP14 was a negative regulator of type I IFN antiviral signaling. To further verify the role of USP14, we knocked down cellular USP14 using siRNAs at both the mRNA and protein levels, and siRNA#2 targeting USP14 showed better knockdown effects (Fig. 6D and E). In contrast, knockdown of USP14 markedly enhanced the phosphorylation of TBK1 and IRF3 induced by SeV infection (Fig. 6F). Concurrently, lower USP14 expression led to a significant increase in ubiquitinated RIG-I, specifically K63-linked, rather than K48-linked, ubiquitination (Fig. 6G). All of these results were consistent with one previous research (22). Collectively, we verified that USP14 indeed removed RIG-I K63-linked ubiquitination to block type I IFN antiviral response.

### miR-30a, by downregulating USP14, enhances RIG-I K63-linked ubiquitination to exert antiviral effects

**Fig 7:**
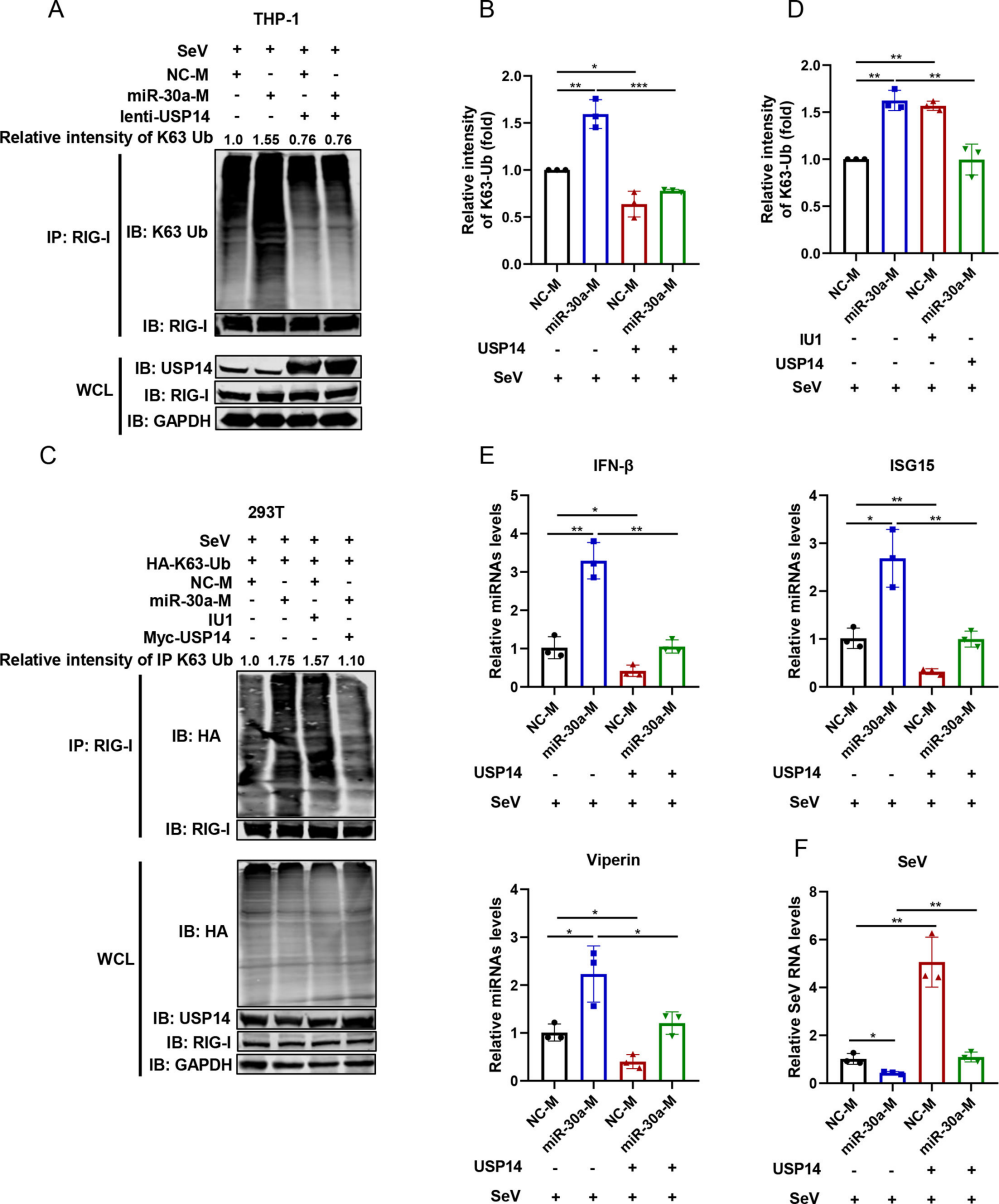
miR-30a, by downregulating USP14 expression, increases RIG-I K63-linked ubiquitination to exert antiviral effects. (**A and B**) NC-M or miR-30a-M were transfected into THP-1 cells for 24 h and infected with lentivirus expressing USP14 for another 24 h. After SeV infection (100 HAU/mL) for 12 h, the endogenous K63-linked RIG-I ubiquitination was determined via immunoblot analysis (**A**). The relative intensities of RIG-I K63-linked ubiquitination expression levels were quantified from three replicates using the Image Studio V5.2 (**B**). (**C and D**) HA-tagged K63-Ub and NC-M or miR-30a-M were co-transfected into HEK293T cells together with Myc-tagged USP14 or empty vector for 48 h and infected with SeV for 12 h. One of the cell samples was pretreated with IU1 inhibitors (100 mM) 24 h before collection. The exogenous K63-linked ubiquitination of RIG-I was detected via immunoblot analysis (**C**), and the relative intensities of RIG-I K63-linked ubiquitination expression levels were quantified from three replicates using the Image Studio V5.2 (**D**). (**E and F**) THP-1 cells were transfected with NC-M or miR-30a-M for 24 h and infected with lentivirus expressing USP14 for another 24 h, followed by SeV infection for 12 h. Then, the relative mRNA expression levels of IFN-β and selected ISGs (ISG15 and Viperin) (**E**) as well as cellular SeV RNA level (**F**) were determined by qRT-PCR analysis. Data are presented as mean ± SD and are representative of three independent experiments. The statistical significance was carried out using a two-tailed Student’s *t*-test. *P < 0.05, **P < 0.01.

miR-30a targets and inhibits USP14 expression, whereas USP14 removed K63-linked ubiquitination from RIG-I. Therefore, we further investigate whether miR-30a modulates RIG-I-mediated antiviral signaling by directly regulating USP14 expression. Immunoblot assay showed that miR-30a increased both endogenous (Fig. 7A, lane 2) and exogenous (Fig. 7C, lane 2) K63-linked ubiquitination of RIG-I, while USP14, as a negative regulator of IFN antiviral signaling, significantly suppressed miR-30a-induced enhancement of endogenous (Fig. 7A, lane 3, 4) and exogenous (Fig. 7C, lane 4) K63-linked ubiquitinated RIG-I. Moreover, similar to miR-30a overexpression, USP14 inhibitor IU1 enhanced RIG-I K63-linked ubiquitination due to the inhibition of USP14 enzyme activities (Fig. 7C, lane 3). Correspondingly, miR-30a promoted IFN-β and downstream ISG production (Fig. 7E, lane 2), thereby inhibiting cellular SeV replication (Fig. 7F, lane 2). However, in the presence of USP14, the increase of IFN-β and ISG production induced by miR-30a was impaired (Fig. 7E, lane 4), thereby promoting SeV replication compared to miR-30a (Fig. 7F, lane 4). In summary, we concluded that miR-30a, by downregulating USP14 expression, enhanced RIG-I K63-linked ubiquitination, leading to an intensive activation of immune response to exert antiviral effects.

## DISCUSSION

**Fig 8:**
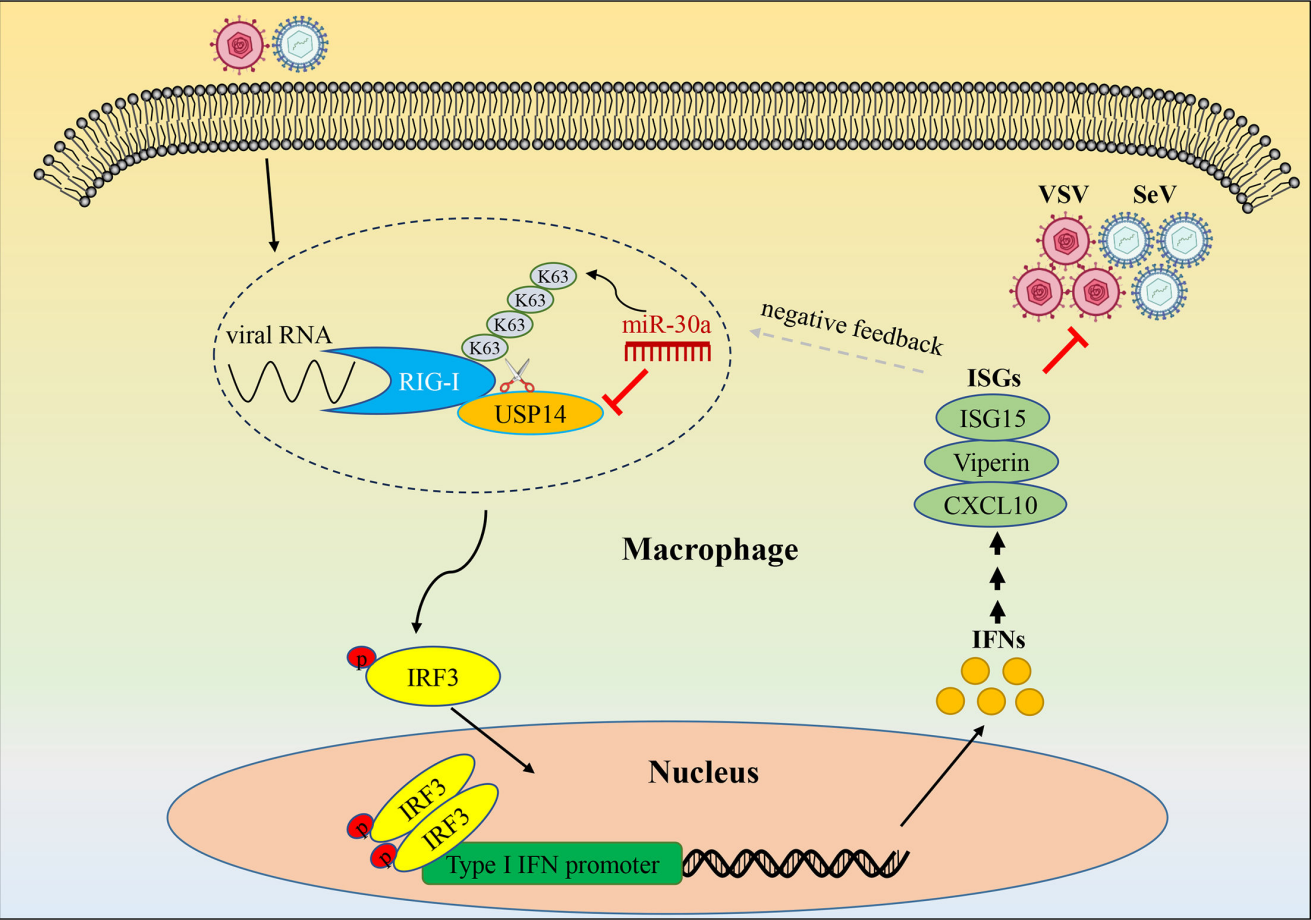
Schematic diagram of miR-30a regulating type I IFN antiviral response by targeting USP14 in macrophages. SeV or VSV infection activates RIG-I-mediated innate immune response. USP14 removes K63-linked ubiquitination of RIG-I to impair IFN signaling, whereas miR-30a binds with 3′UTR mRNA to inhibit USP14 expression. Consequently, miR-30a promotes RIG-I-mediated signaling to exert broad-spectrum antiviral effects. In return, the activation of type I IFN signaling suppresses miR-30a expression to avoid the disordered activation. Black lines represent “enhancing,” red lines represent “blocking,” and gray dotted lines represent “negative feedback.”

Currently, the major antiviral strategies include preventative vaccination and therapeutic drug interventions. However, it is urgent to develop novel broad-spectrum antiviral strategies due to the accelerated evolution of viruses and the emergence of drug-resistant strains. As is known to us, IFN is an effective broad-spectrum antiviral against various viruses through different mechanisms (28). On the other hand, viruses can evade IFN response by impairing or disrupting factors involved in IFN signaling. In this study, we demonstrated that miR-30a specifically inhibited USP14 expression by binding to the USP14 3′UTR, thereby promoting RIG-I K63-linked ubiquitination and enhancing RIG-I-mediated type I IFN antiviral signaling against viral infection (Fig. 8). Our findings reveal a novel regulatory mechanism that serves as a significant element of the host defense system against viral infections.

miRNAs are non-coding small RNA that serve as important regulators in the host cells, which have been proven to contribute to the regulation of various viral infections, either by binding with the viral genome or by modulating the host antiviral immune defenses. The classic report of miRNA targeting the viral genome was known as miR-122 binding with the HCV RNA to promote its replication (29). On the other hand, up to now, many miRNAs have been identified as regulators in type I IFN signaling. For instance, miR-33/33* inhibited the activation of MAVS through AMPK in the antiviral innate immunity (30); miR-200b-3p suppressed type I IFN antiviral signaling by directly targeting TBK1 (31), whereas miR-223 promoted type I IFN production by targeting forkhead box protein O3 (FOXO3) (32). Herein, we demonstrated that miR-30a was a positive regulator involved in type I IFN regulation.

The miR-30 family members have been demonstrated to be involved in the regulation of multiple processes, including development, apoptosis, and autophagy (33–35). Several studies have reported that miR-30a was involved in the regulation of viral life cycles. But the regulatory relationships between miR-30a and different viruses vary widely. One previous study has reported that miR-30a inhibited IAV infection through reinforcing type I IFN response by targeting SOCS1, SOCS3, and NEDD4 in A549 cells (16). Another research also demonstrated that miR-30a enhanced IFN-I antiviral activity by directly binding with SOCS1 and SOCS3 in ST cells, thereby suppressing transmissible gastroenteritis virus (TGEV) infection (17). In contrast, miR-30a was identified as a negative regulator of type I IFN signaling in some other studies. For instance, miR-30a has been shown to facilitate coxsackievirus B3 (CBV) replication through blocking IFN production via targeting TRIM25 in HeLa cells (15). In another study, miR-30a suppressed type I interferon signaling by targeting myeloid differentiation factor 88 (MyD88), thereby promoting enterovirus 71 (EV71) replication (36). It is likely that the miR-30a regulatory role varies upon various virus infections in different cells. However, up to now, how miR-30a regulates type I IFN signaling in immune cells is unclear. Therefore, in this study, we tried to explore the regulatory roles of miR-30a on type I IFN signaling in macrophages.

Notably, we observed a significant decrease in miR-30a expression induced by SeV or VSV infection in THP-1 cells. In contrast, one previous study showed that VSV infection or poly(I:C) treatment has no influence on miR-30a expression in A549 cells (16). These contradictory results indicate that the expression of miR-30a regulated by type I IFN signaling varies with different cell types, and we believe that the results from immune cells are more convincing. However, all these researches demonstrated that miR-30a expression was downregulated by various viral infections, although miR-30a exerts diverse regulatory mechanisms for different viruses (14–17). We speculate that the decrease of miR-30a expression is dependent on the activation of type I IFN immune response triggered by viral infections. Furthermore, it has also been reported that miR-30c was upregulated by porcine reproductive and respiratory syndrome virus (PRRSV) in porcine alveolar macrophages (37), indicating that the expression of various miR-30 family members can be differentially regulated by different stimulants.

RIG-I-mediated antiviral signaling is largely regulated by RIG-I ubiquitination, while RIG-I ubiquitination is also regulated by some miRNAs. Our recent research verified that miR-26a enhanced RIG-I ubiquitination by targeting USP15 (38). Another earlier research also showed that miR-202-5p hindered TRIM25-mediated ubiquitination of RIG-I, leading to attenuated activation of IFN production (39). Similarly, in this study, we demonstrated that miR-30a modulated type I IFN production by enhancing RIG-I K63-linked ubiquitination. With this in mind, aiming to elucidate the specific mechanism of miR-30a regulating RIG-I ubiquitination, we selected some potential miR-30a target genes related to ubiquitin regulation for further screening via luciferase reporter assay. Consequently, we screened and identified USP14 as the target of miR-30a for the first time. USP14 belongs to the USP family member of DUBs superfamily, which has been shown to be involved in the regulation of neuron diseases (26), cancer (40), as well as viral proliferation (41). Previous studies have reported that USP14 interacted with RIG-I and removed K63-linked ubiquitination from RIG-I, thereby negatively regulating type I IFN antiviral response (22). Consistently, knockdown of USP14 by siRNA or activity inhibition of USP14 by IU1 both significantly augmented RIG-I-mediated type I IFN signaling in our study. Furthermore, USP14 significantly impaired miR-30a-induced increase of RIG-I K63-linked ubiquitination as well as IFN-β or ISG production, further indicating that miR-30a plays a regulatory role by targeting USP14. Therefore, we concluded that miR-30a enhances type I IFN antiviral signaling to inhibit viral infection through the miR-30a-USP14-RIG-I ubiquitination axis.

In summary, this research revealed a novel regulatory mechanism of miR-30a on type I IFN antiviral response. miR-30a exerts antiviral effects by facilitating SeV- or VSV-triggered type IFN production through inhibition of USP14 expression, thereby leading to an increase of RIG-I K63-linked ubiquitination. We also observed that robust activation of type I IFN responses could, in turn, impair miR-30a expression, suggesting the critical roles of inherent negative feedback loops. These observations enriched the network between miRNAs and innate immune responses and provided new insights into the roles of host miRNAs in defense against viral infections.

## MATERIALS AND METHODS

### Cell culture and antibodies

THP-1 cells were cultured in 1640 medium (Bio-Channel) supplemented with 10% fetal bovine serum (FBS) (Bio-Channel) and induced into macrophages by treatment with phorbol 12-myristate 13-acetate (PMA, 200 ng/mL) (MedChemExpress, MCE) for 24 h. HEK293T cells were grown in DMEM medium (Bio-Channel) and supplemented with 10% FBS. All types of cells were maintained in a humidified incubator at 37°C with 5% CO2.

The monoclonal antibodies (mAbs) against p-TBK1 (Ser172, 5483T), p-IRF3 (Ser386, 37,829T), WT-Ub (20326S), K48-Ub (8081S), and K63-Ub (5621S) were purchased from Cell Signaling Technology (CST). The mAbs against Flag (M20008) and HA (M20003) were obtained from Abmart. The polyclonal antibodies (pAbs) against TBK1 (28397-1-AP), IRF3 (11312-1-AP), USP14 (14517-1-AP), RIG-I (20566-1-AP), Myc (16286-1-AP), and GAPDH (10494-1-AP) were acquired from Proteintech.

### Viral infection and virus titration

Cells (THP-1 and HEK293T) were infected with SeV at a final concentration of 100 hemagglutinin units per mL (HAU/mL) or VSV at a MOI of 0.1 for the indicated times. The cell culture supernatants were collected for the virus titration via TCID_50_ assay in Vero cells as described previously. In brief, Vero cells were seeded into 96-well plates (1 × 10^4^ per well), followed by inoculation with tenfold sequential dilutions of each sample for three days. Then, the viral titers were calculated based on three replicates and expressed as log10 TCID_50_/mL using the Reed-Muench method.

### Plasmids and reagents

Flag-tagged RIG-I, HA-tagged WT-Ub, K48-Ub, and K63-Ub plasmids (all lysine in the ubiquitin are mutated into alanine except for K48 or K63 sites) were stored in the laboratory. The partial 3′UTR sequences of USP48, USP45, USP22, USP2, USP14, CYLD, TRIM13, TRIM27, and NEDD4 that bind miR-30a were cloned into the pmiRGLO vector (a dual-luciferase expression vector for both firefly and Renilla luciferase). The 3′UTR sequences of USP14 binding with the miR-30a seed region in the wild-type constructs were mutated. Myc-tagged USP14 was gifted from Prof. Bo Zhong (Wuhan University). USP14 lentiviral plasmids were constructed by cloning USP14 coding sequences (CDS) into lentiviral vectors. USP14 inhibitors IU1 (HY-13817) were purchased from MCE. The double-stranded RNA mimic poly(I:C)-LMW (tlrl-picw) was acquired from InvivoGen.

### Transfection of siRNAs or miRNAs

siRNA, miRNA mimics, and inhibitors were synthesized by GenePharma. The sequences were listed as follows: NC mimics, 5′-UUCUCCGAACGUGUCACGUTT-3′ (sense) and 5′-ACGUGACACGUUCGGAGAATT-3′ (anti-sense); miR-30a mimics, 5′-UGUAAACAUCCUCGACUGGAAG-3′ (sense) and 5′-UCCAGUCGAGGAUGUUUACAUU-3′ (antisense); NC inhibitors, 5′-CAGUACUUUUGUGUAGUACAA-3′; miR-30a inhibitors: 5′-CUUCCAGUCGAGGAUGUUUACA −3’; siUSP14#1, 5′-GACAGAAAGUUAUGGUGAAAG-3′; and siUSP14#2, 5′-AGUUCUUAAGGAUGUUAAAUU-3′. siRNAs or miRNAs were transfected into cells using jetPRIME (Polyplus) following the manufacturer’s protocol. In brief, siRNAs (60 nM), miRNA mimics (80 nM), and inhibitors (120 nM) were mixed with jetPRIME in opti-DMEM for 15 min and transfected into cells for 48 h.

### Luciferase reporter assay

#### 3′UTR luciferase reporter assays

The above-constructed wild-type or mutant pmiRGLO-3′UTR plasmids were transfected into cells together with miR-30a mimics or inhibitors for 48 h, respectively. Then, the relative luciferase activities were determined by calculating the ratio of firefly to Renilla luciferase activities using the dual-luciferase reporter (DLR) assay system (Vazyme, DL101-01).

#### IFN-β promoter luciferase reporter assays

IFN-β luciferase (IFN-β-Luc) promoter and pRL-TK plasmid (expressing the Renilla luciferase as an internal control) were co-transfected into HEK293T cells together with USP14 expression plasmid or empty vector for 36 h, followed by SeV infection for 12 h. The activation of the IFN-β promoter was determined by calculating the ratio of firefly to Renilla luciferase activities.

### Quantitative real-time PCR (qRT-PCR)

#### Quantitation of cellular genes or viral copies

Total cellular RNA was extracted using Trizol reagent. The first-strand cDNA synthesis was carried out by reverse transcription with random primers using HiScript II Q RT SuperMix (Vazyme, R223-01). qPCR was performed using ChamQ SYBR Green Master Mix (Vazyme, Q311-02) via LightCycler 480 system (Roche). The relative mRNA expression levels of various genes were determined by normalizing to GAPDH expression via the 2^-△△^*^CT^* threshold method. The primers used are listed in Table 1.

**TABLE 1:** Primer sequences for qRT-PCR of mRNAs

Primers	Sequences (5′−3′)
qSeV-F	GACAGATGAGATATCGTGGATGG
qSeV-R	TCTAAGCTCAGATTAGCCCTTGT
qVSV-F	GAAAGGGAACTGTGGGATGA
qVSV-R	GAACACCTGAGCCTTTGAGC
qIFN-β-F	ATGACCAACAAGTGTCTCCTCC
qIFN-β-R	GCTCATGGAAAGAGCTGTAGTG
qISG15-F	CTCTGAGCATCCTGGTGAGGAA
qISG15-R	AAGGTCAGCCAGAACAGGTCGT
qViperin-F	CCAGTGCAACTACAAATGCGGC
qViperin-R	CGGTCTTGAAGAAATGGCTCTCC
qGAPDH-F	GTCTCCTCTGACTTCAACAGCG
qGAPDH-R	ACCACCCTGTTGCTGTAGCCAA
qUSP14-F	AGGTCATTATGTATCATGGGTG
qUSP14-R	ATTTCAACTCTGCGAGGC

#### Quantitation of miRNAs

Total cellular RNA extracted by Trizol was reverse transcribed with miR-30a stem-loop RT primer and U6 reverse primer using miRNA 1st Strand cDNA Synthesis Kit (Vazyme, MR101-01). Then, qPCR was performed as described above. The relative expression levels of cellular miRNAs were normalized to U6 expression via the 2^-△△*CT*^ threshold method. The primers used for stem-loop qPCR are listed in Table 2.

**TABLE 2:** Primer sequences for qRT-PCR of miRNAs

Primers	Sequences (5′−3′)
miR-UR-R	CAGTGCAGGGTCCGAGGTAT
U6-F	CTCGCTTCGGCAGCACA
U6-R	AACGCTTCACGAATTTGCGT
RT-let-7a-5p	GTCGTATCCAGTGCAGGGTCCGAGGTATTCGCACTGGATACGACAACTATA
let-7a-5p-F	CCAGTGAGGTAGTAGGTTG
RT-miR-30a-5p	GTCGTATCCAGTGCAGGGTCCGAGGTATTCGCACTGGATACGACCTTCCA
miR-30a-5p-F	GCTGTAAACATCCTCGACTGGAAG
RT-miR-122-5P	GTCGTATCCAGTGCAGGGTCCGAGGTATTCGCACTGGATACGACCAAACAC
miR-122-5P-F	CCAGTGGAGTGTGACAATG
RT-miR-125b-5P	GTCGTATCCAGTGCAGGGTCCGAGGTATTCGCACTGGATACGACTCACAAG
miR-125b-5p-F	GGAATCCCTGAGACCCTAA
RT-miR-155	GTCGTATCCAGTGCAGGGTCCGAGGTATTCGCACTGGATACGACCCCC
miR-155-F	CTCGTGGTTAATGCTAAT
RT-miR-181a-5p	GTCGTATCCAGTGCAGGGTCCGAGGTATTCGCACTGGATACGACACTCACC
miR-181a-5p-F	CCGGAACATTCAACGCTGTC
RT-miR-196a-5p	GTCGTATCCAGTGCAGGGTCCGAGGTATTCGCACTGGATACGACCCCAACA
miR-196a-5p-F	CCGGTAGGTAGTTTCATGT
RT-miR-199a-3p	GTCGTATCCAGTGCAGGGTCCGAGGTATTCGCACTGGATACGACTAACCAA
miR-199a-3p-F	ACAGTAGTCTGCACA
RT-miR-210-5p	GTCGTATCCAGTGCAGGGTCCGAGGTATTCGCACTGGATACGACCAGTGTG
miR-210-5p-F	ATTAAGCCCCTGCCCACCG
RT-miR-448	GTCGTATCCAGTGCAGGGTCCGAGGTATTCGCACTGGATACGACATGGGAC
miR-448-F	CCGGTTGCATATGTAGGAT

### Immunoblot analysis

Cells were lysed with RIPA or NP-40 lysis buffer (Beyotime) supplemented with a protease inhibitor cocktail (VICMED, China). The cell lysates were sonicated and subjected to sodium dodecyl sulfate gel electrophoresis (SDS-PAGE). Then, the protein was electrically transferred into PVDF membranes (Milipore) and blocked with 5% skim milk for 1 h. Then, the membranes were incubated with a specific primary antibody at 4°C overnight, followed by incubation with IRDye 680CW or 800CW secondary antibodies (LI-COR). Finally, the protein bands were visualized using Odyssey CLx Image Studio (LI-COR).

### Overexpression of USP14 by lentivirus infection

In order to overexpress USP14 in THP-1 cells, the lentivirus expressing USP14 was obtained via three-plasmid packaging system. In brief, the recombinant lentiviral vectors expressing USP14 were transfected into HEK293T cells together with pCMVR8.74 and pMD2G plasmids for 72 h. Then, the supernatants were collected and concentrated by ultracentrifugation. Subsequently, THP-1 cells were infected with lentiviruses expressing USP14 using polybrene.

### Ubiquitination assay

Cells were treated with MG132 (HY-13259, MCE) for 4 h before collection and lysed with NP-40 lysis buffer. Then the lysates underwent immunoprecipitation with anti-RIG-I or anti-Flag antibodies overnight at 4°C and were incubated with magnetic beads for 8 h at 4°C. The beads were recovered and washed five times with PBS buffer and resuspended in 2 × loading buffer. The endogenous or exogenous ubiquitinated RIG-I was determined by immunoblot analysis using the indicated antibodies.

### Statistical analysis

Statistical analysis was performed via GraphPad Prism 8.0 software, and the data were presented as mean ± SD. The statistical significance was assessed using a two-tailed Student’s *t*-test (NS [not significant] >0.05, *P < 0.05, **P < 0.01, ***P < 0.001).

## Data Availability

The raw data supporting this paper are available from the corresponding author on reasonable request.
